# Acral Amelanotic Melanoma Mimicking a Foot Ulcer

**DOI:** 10.7759/cureus.26615

**Published:** 2022-07-06

**Authors:** Harrison J Shawa, Marat Kazak, Sara Dahle, Joshua M Schulman

**Affiliations:** 1 Dermatology Section, VA Northern California Healthcare System, Sacramento, USA; 2 Podiatry Section, VA Northern California Healthcare System, Sacramento, USA; 3 Dermatology, University of California Davis, Sacramento, USA

**Keywords:** diagnostic dilemma, chronic wound, acral amelanotic melanoma, acral melanoma, amelanotic melanoma

## Abstract

Acral amelanotic melanoma can be difficult to diagnose and is often clinically aggressive. The present report describes a case of an acral amelanotic melanoma presenting as a non-healing wound after mimicking a plantar wart for two years. The decision to biopsy a borderline-suspicious lesion on the lower extremity in an elderly individual must be weighed carefully, as lower extremity biopsy carries a risk of poor wound healing and other complications. We discuss clinical and epidemiologic features that can assist in deciding when to perform a biopsy in this setting and can improve the early detection of acral amelanotic melanoma.

## Introduction

Amelanotic melanoma, accounting for less than 2% of melanomas, lacks typical clinical features of melanoma and mimics other lesions, frequently resulting in initial misdiagnosis and treatment delays and contributing to a poorer prognosis compared to conventional melanoma [1].

Amelanotic melanoma affects both men and women and, on average, affects older individuals than conventional melanoma, with an average age at diagnosis of 62 years [1]. Although some risk factors overlap with melanoma, individuals with amelanotic melanoma are more likely to have red hair, freckles, or sunburn easily than patients with pigmented melanoma [2]. Other risk factors include more than 10 large nevi, plantar nevi, and a history of a penetrating foot injury or a previous amelanotic melanoma [2].

When occurring on acral sites, amelanotic melanoma may mimic a variety of benign entities, including verrucae, calluses, poromas, hematomas, foreign bodies, fungal infections, blisters, ulcers, and pyogenic granulomas [3-6]. We herein report a case of an acral amelanotic melanoma presenting as an ulcer preceded by a callus on a non-weight-bearing aspect of the foot that was initially diagnosed as a wart.

## Case presentation

**Figure 1:**
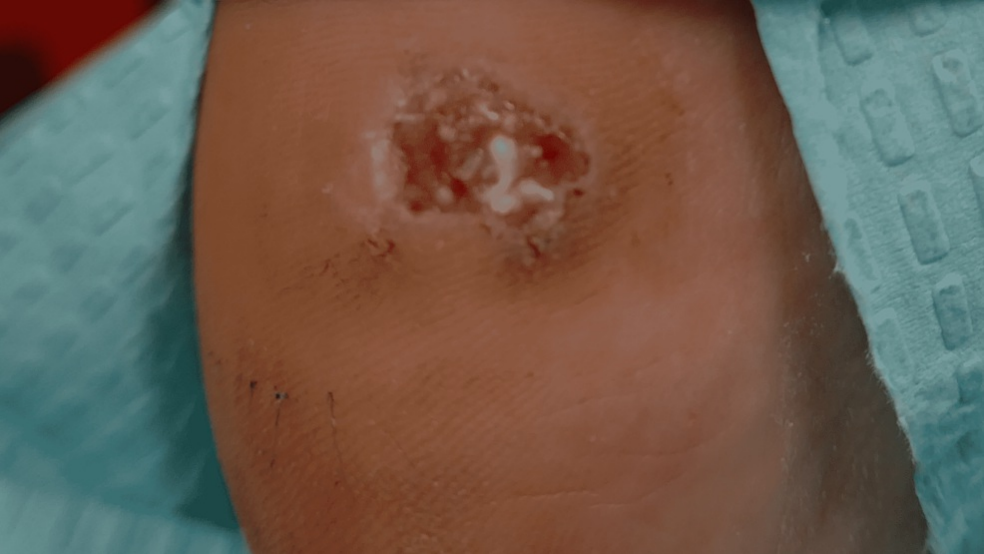
Clinical features prior to initial biopsy. A 1.2 cm x 0.6 cm ulcer with a granular wound bed and hyperkeratotic, irregular margins.

**Figure 2:**
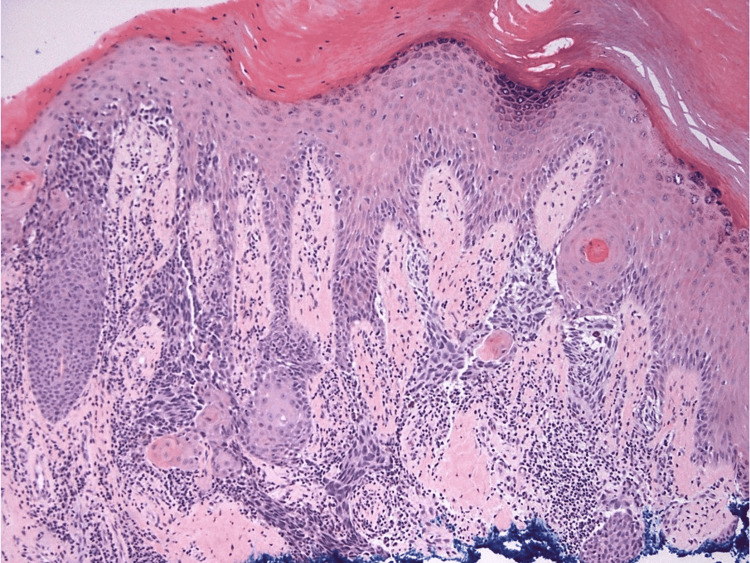
Nests of atypical melanocytes are arrayed in confluence along the basal layer underlying a papillated epidermal surface. Melanocytes extend deeply along adnexal epithelium and are present within the underlying dermis (hematoxylin & eosin, original magnification 100x).

**Figure 3:**
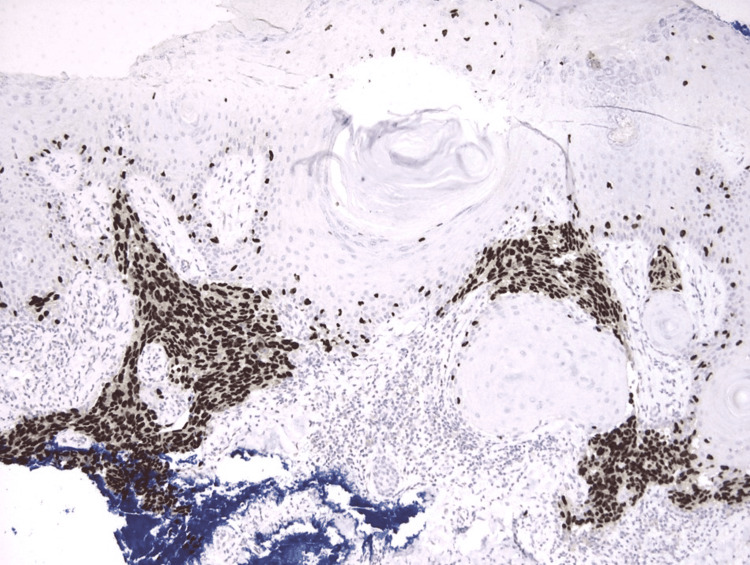
Sox-10 immunostaining highlights the large nests of melanocytes and also labels scattered melanocytes above the basal layer (original magnification 100x).

A 67-year-old man with a past medical history of diabetes mellitus presented to the podiatry clinic with a four-month history of a painful, nonhealing wound on the medial aspect of the right heel which had been treated unsuccessfully with topical mupirocin. Prior to wound formation, a hyperkeratotic lesion had been present for two years, which the patient believed was a wart. The lesion had several episodes of traumatic excoriation, bleeding, and then partial healing. Physical examination revealed a 1.2 cm x 0.8 cm, irregularly shaped, draining ulcer with a granular wound bed on the medial right heel (Figure 1). The neurovascular examination was normal and the musculoskeletal exam revealed arthritis of the toes. The patient was treated with local wound care and custom-fitting shoes, with instructions to return in two weeks to assess for improvement. At the follow-up visit, given the lack of clinical improvement and atypical location, the lesion was biopsied revealing a melanoma. Following the biopsy, wide local excision with 2 cm margins and sentinel lymph node biopsy were performed, confirming melanoma, stage pT2b, with a Breslow depth of 1.5 mm (Figure 2), and negative margins and lymph nodes (Figure 3). 

The surgical wound, 4.0 cm x 4.5 cm, was treated with negative pressure wound therapy (NPWT) at 125 mmHg continuously with thrice-weekly dressing changes. The wound healed at an average rate of 1.96 cm2/week and with no signs of infection. However, five weeks postoperative, the area surrounding the inguinal lymph node biopsy site became erythematous, warm, and indurated. Mild cellulitis, related to manipulation of the incision site, was suspected, and the patient was treated and improved after a seven-day course of oral cephalexin. After six weeks of NPWT, the wound edges appeared macerated, so the patient was transitioned to other wound care modalities. Over the course of 13 weeks, these included silver alginate, Durafiber® (Smith & Nephew, Andover, MA), Medihoney® (Integra Lifesciences, Princeton, NJ), and Iodosorb® (Smith & Nephew, Andover, MA), ultimately resulting in the wound’s complete epithelialization. At the last available follow-up, nine months post-biopsy, the patient remained free of recurrence or clinical evidence of metastasis.

## Discussion

Acral melanoma accounts for less than 3%-15% of all melanoma diagnoses [4]. While 2%-20% of melanomas are amelanotic or hypomelanotic, with an annual U.S. incidence of 1.8 per 1,000,000 persons, up to 30% of acral melanomas lack pigmentation [1, 7]. Early lesions lack the typical diagnostic features of melanoma and may present as asymmetric pink nodules with color uniformity, indistinct borders, and a slightly pigmented periphery, progressing to ulceration, a red plaque, or a granulomatous nodule. Regardless of pigmentation, acral melanoma has a higher misdiagnosis rate relative to other locations. Around 10%-54% are initially misdiagnosed, which, alongside acral melanoma’s inherent aggressiveness, likely results in a more advanced stage at diagnosis and worse prognosis [8]. Early detection of acral melanoma is essential, as five- and ten-year melanoma-specific survival rates are 80.3% and 67.5%, respectively, much lower than those of all cutaneous melanomas, which are 91.3% and 87.5%, respectively [8].

Given the clinical diagnostic difficulties of acral and amelanotic melanomas individually, acral amelanotic melanomas present a considerable challenge, particularly when mimicking a common, benign lesion, such as a wart. Clinically, viral warts often display black dots, colloquially known as ‘the roots’ of the wart, which arise due to capillary thrombosis. Hypomelanotic melanoma with focal pigmented areas may mimic these dots, or alternatively, when more generalized, subcutaneous hemorrhage [4]. Furthermore, these lesions tend to be treated with multiple rounds of cryotherapy, altering local morphology and potentially masking “red flag” symptoms.

While amelanotic melanoma is a universal clinical diagnostic challenge, dermoscopy can be helpful, potentially revealing features such as white structureless zones, milky-red areas, or arborizing, linear looped, or linear irregular vessels, indicating an increased likelihood of amelanotic melanoma [9]. However, it is not perfect, as amelanotic acral melanoma may be dermoscopically featureless [2]. It has been suggested that all plantar warts in elderly individuals should undergo, or at least be considered for, excisional or incisional biopsy to rule out amelanotic melanoma [4]. However, a biopsy of the foot carries its own risks, including delayed healing, particularly in elderly individuals with impaired mobility or vascular comorbidities such as diabetes, venous stasis, or peripheral arterial disease. In these patients, biopsy should be reserved for lesions with high-risk features such as itching, bleeding, treatment resistance, growth, or associated nail deformation [4]. Although it may appear to reduce the risk of complications, superficial shave biopsy is not recommended, as it may not achieve adequate depth to detect melanoma [4].

Non-invasive melanoma adhesive gene expression testing, which can distinguish malignant pigmented lesions from benign ones with approximately 95% sensitivity and 91% specificity [10], is an additional tool that may prove to be effective in the detection of acral amelanotic melanoma in the future. However, nonpigmented and acral lesions cannot yet be analyzed with this method, as sufficient RNA cannot be obtained from palms or soles, and the method is only validated in pigmented lesions [11]. If optimized for these lesions, adhesive gene expression testing would minimize the concern of morbidity associated with lower extremity biopsy, likely resulting in more judicious biopsy practices of borderline-suspicious lesions. Perhaps future studies will validate it for nonpigmented lesions suspicious of amelanotic melanoma, as well as ulcerated or pared-down hyperkeratotic plantar lesions. 

## Conclusions

Given the high mortality rate of amelanotic melanoma, early diagnosis is essential. Utilizing both the epidemiologic factors mentioned above and clinical history of treatment resistance, atypical features, and distribution unexplained by biomechanics, may increase the clinical suspicion of amelanotic melanoma and can help the clinician weigh the decision to biopsy. Ultimately, awareness of anorectal malignant melanoma (AMM) as “the great masquerader” may improve early detection and clinical outcomes of this deadly melanoma variant.
